# High-Throughput Screening for the Identification of New Therapeutic Options for Metastatic Pheochromocytoma and Paraganglioma

**DOI:** 10.1371/journal.pone.0090458

**Published:** 2014-04-03

**Authors:** Alessio Giubellino, Uma Shankavaram, Petra Bullova, Jan Schovanek, Yaqin Zhang, Min Shen, Nikita Patel, Abdel Elkahloun, Min-Jung Lee, Jane Trepel, Marc Ferrer, Karel Pacak

**Affiliations:** 1 Program in Reproductive and Adult Endocrinology, Eunice Kennedy Shriver National Institute of Child Health & Human Development, NIH, Bethesda, Maryland, United States of America; 2 National Center for Advancing Translational Sciences, NIH, Rockville, Maryland, United States of America; 3 National Human Genome Research Institute, NIH, Bethesda, Maryland, United States of America; 4 Medical Oncology Branch, National Cancer Institute, NIH, Bethesda, Maryland, United States of America; 5 Radiation Oncology Branch, National Cancer Institute, NIH, Bethesda, Maryland, United States of America; 6 Department of Molecular Medicine, Institute of Virology, Slovak Academy of Sciences, Bratislava, Slovak Republic; 7 Department of Internal Medicine III – Nephrology, Rheumatology and Endocrinology, University Hospital of Olomouc, Czech Republic; Albert-Ludwigs-University, Germany

## Abstract

Drug repurposing or repositioning is an important part of drug discovery that has been growing in the last few years for the development of therapeutic options in oncology. We applied this paradigm in a screening of a library of about 3,800 compounds (including FDA-approved drugs and pharmacologically active compounds) employing a model of metastatic pheochromocytoma, the most common tumor of the adrenal medulla in children and adults. The collection of approved drugs was screened in quantitative mode, testing the compounds in compound-titration series (dose-response curves). Analysis of the dose-response screening data facilitated the selection of 50 molecules with potential bioactivity in pheochromocytoma cells. These drugs were classified based on molecular/cellular targets and signaling pathways affected, and selected drugs were further validated in a proliferation assay and by flow cytometric cell death analysis. Using meta-analysis information from molecular targets of the top drugs identified by our screening with gene expression data from human and murine microarrays, we identified potential drugs to be used as single drugs or in combination. An example of a combination with a synergistic effect is presented. Our study exemplifies a promising model to identify potential drugs from a group of clinically approved compounds that can more rapidly be implemented into clinical trials in patients with metastatic pheochromocytoma or paraganglioma.

## Introduction

Pheochromocytoma (PHEO) is a rare neuroendocrine tumor that develops in the adrenal medulla and represents the most common tumor in this location in children and adults [1]. Although often sporadic, PHEO or paraganglioma (an extra-adrenal tumor; PGL) may present in several familial syndromes, and certain subtypes are particularly prone to malignancy [2]–[4]. Studies of these familial syndromes have enormously improved our understanding of the underlying genetic basis of the disease, and several molecular pathways have been “put on the map” [3], [5], [6]. This knowledge is helping in charting a new era of therapeutic strategies for the disease. However, at the moment there is no treatment for metastatic PHEO/PGL that is either curative or capable of inducing durable responses [7], [8]. Several therapeutic options relieve patient symptoms and signs and decrease tumor burden, but relapses often occur, resulting in ultimate death [7], [9], [10].

From an industry perspective, drug development programs for rare (“orphan”) diseases such as PHEO/PGL (approximately 1,000 new cases are diagnosed in the US each year) are less appealing because of the low return on investment. Thus, alternative approaches must be sought to discover novel therapeutic options for these tumors. One potential strategy is to “recycle” drugs that have been approved for use in the treatment of other diseases, a strategy known as drug repurposing or repositioning [11]–[13]. Drugs that received regulatory approval have already proven to be safe and effective for a particular disease. In addition, historical information regarding their pharmacokinetics, pharmacodynamics and long-term side effects in a large population is available, which makes repurposing for other diseases less time-consuming and expedites their introduction into clinical trials that are very much needed. Of particular concern are patients carrying mutations in the SDHB gene, as these patients are more prone than other patients to develop more aggressive and metastatic disease [14], [15].

In the present study we identified and validated new therapeutic options for PHEO/PGL by screening the NIH Chemical Genomic Center (NCGC) Pharmaceutical Collection (NPC), a large library of clinically approved drugs [13], [16]. We then selected representative compounds from the top 50 active drugs from the initial screen and conducted further validation. We finally performed a meta-analysis using data from human and murine PHEO/PGL microarrays to attempt to identify molecular targets and pathways that can be affected by these drugs. We demonstrated, with an example of a synergistic drug combination, the potential benefit of our study in selecting drugs for combined therapies. Finally, we have introduced a new group of potential drugs from clinically approved compounds that could more rapidly be implemented into clinical trials in patients with metastatic PHEO/PGL.

## Materials and Methods

### Cell lines and reagents

We used the following cell lines, which represent the only available permanent PHEO cell lines available to the scientific community and include a range of models of PHEO. The rat PHEO cell line PC12, developed in 1976, has a MAX gene deletion that has been recently discovered in a human PHEO kindred [17]. The mouse MPC cell line, derived from *NF1* knock-out mice, represents a well differentiated model, with a behavior very similar to most slow-growing PHEO/PGL; interestingly, recent evidence points to NF1 loss of function as a frequent occurrence in sporadic PHEO [18]. Finally, the MTT cell line, which is rapidly growing and derived from the liver metastases of MPC cells, is a model related to some more aggressive human PHEO/PGL, with accelerated metastatic behavior [19]. The mouse PHEO cell lines MPC and MTT were maintained in DMEM supplemented with 10% fetal bovine serum (FBS) and 5% horse serum (Gibco), antibiotic/antimycotic. The rat PHEO cell line PC12 was maintained in DMEM supplemented with 10% FBS and antibiotic/antimycotic. Cells were grown until 80% confluence, then cells were detached using 0.05% trypsin/EDTA, incubated for 3 minutes at 37°C and resuspended and counted to obtain the desired concentration before experiments.

### Quantitative high-throughput proliferation assay summary and protocols for MTT cells

Cell viability after compound treatment was measured using a luciferase-coupled ATP quantitation assay (CellTiter-Glo, Promega) in MTT cells. The change of intracellular ATP content indicates the number of metabolically competent cells after compound treatment. MTT cells were harvested from T225 flasks and resuspended in DMEM medium with 5% FBS and 1% horse serum. Then 5 µl of a suspension of 200,000 cells/ml was dispensed into each well of white, solid bottom, 1536-well tissue culture–treated plates using a Multidrop Combi dispenser. After overnight culture at 37°C with 5% CO_2_, a total of 23 nl of compounds at 8 selected concentrations from the NPC or positive control (10 mM stock of doxorubicin hydrochloride) in DMSO was transferred to each well of the assay plate using a pintool (Kalypsys, San Diego, CA), and the plates were further incubated at 37°C with 5% CO_2_ for 24 or 48 hrs. After that, 4 µl of CellTilter-Glo™ luminescent substrate mix (Promega) was added to each well. The plate was incubated at room temperature for 15 minutes. The plates were measured on a ViewLux plate reader (PerkinElmer) with a clear filter. The final concentration of the compounds in the 5 µl assay volume ranged from 0.5 nM to 46 µM.

### NPC library screening

The NPC consists of 3,826 small molecule compounds, with 52% of the drugs approved for human or animal use by the FDA [16]. The remaining drugs are either approved for use internationally (i.e. in Europe, Canada, or Japan), or are compounds that have been tested in clinical trials. Additional detailed information on the drug library can be found at http://tripod.nih.gov/npc/.

The compounds from the NPC library were prepared as 15 interplate titrations, which were serially diluted 1∶2.236 in dimethyl sulfoxide (DMSO) (Thermo Fisher Scientific, Waltham, MA) in 384-well plates. The stock concentrations of the test compounds ranged from 10 mM to 0.13 µM. The transfer of the diluted compounds from 384-well plates to 1536-well plates was performed using an Evolution P^3^ system (PerkinElmer Life and Analytical Sciences, Waltham, MA). Each treatment plate included concurrent DMSO and positive control wells and concentration-response titrations of controls, all occupying columns 1 to 4. During screening, the compound plates were sealed and kept at room temperature, whereas other copies were maintained at −80°C for storage.

### Data analysis

#### qHTS data analysis and curve fitting

To determine compound activity in the qHTS assay, the titration-response data for each sample were plotted and modeled by a four parameter logistic fit yielding IC_50_ and efficacy (maximal response) values. Raw plate reads for each titration point were first normalized relative to positive control (doxorubicin hydrochloride, 100% inhibition) and DMSO-only wells (basal, 0%). Curve-fits were then classified by the criteria described [20]. Usually the qHTS screen yielded hits with a wide range of potencies and with substantial variation in the quality of the corresponding CRCs (efficacy and number of asymptotes), which included samples associated with shallow curves or single-point extrapolated concentration responses; these were assigned as low-confidence actives. In brief, Class 1.1 and 1.2 were the highest-confidence complete CRCs containing upper and lower asymptotes with efficacies ≥80% and <80%, respectively. Class 2.1 and 2.2 were incomplete CRCs having only one asymptote with efficacy ≥80% and <80%, respectively. Class 3 CRCs showed activity at only the highest concentration or were poorly fit. Class 4 CRCs were inactive, having a curve-fit of insufficient efficacy or lacking a fit altogether.

There were a total of 30 plates in the primary qHTS screen, which included 24 plates corresponding to the NPC library set and 6 DMSO plates. Compounds from the primary qHTS screen were classified into three categories according to the quality of curve fit and efficacy. Actives were compounds in curve class 1.1, 1.2, 2.1 and 2.2 curves with efficacy higher than 60%; inactives were compounds with class 4 curves; and inconclusive included all other compounds including those shallow curves and curves with single point activity.

### Cell proliferation assay

Cell proliferation was determined by the MTT assay (also referred as 3-(4,5-dimethylthiazol-2-yl)-2,5-diphenyltetrazolium bromide assay). MTT cells (15×10^3^) were incubated in 96-well plates for 24 hours in complete medium before the addition of the indicated compound. A solution of 3-(4,5-dimethylthiazol-2-yl)-2,5-diphenyltetrazolium bromide (1 mg/ml; Sigma-Aldrich) was added and plates were incubated at 37°C for 3 hours before measuring absorbance at 562 nm using a Wallac Victor 3 1420 Multilabel plate reader (Perkin Elmer).

### Drug treatment and Western blotting

For PARP cleavage, MTT cells were treated with the indicated dose and concentration of drug for 20 hrs at 37°C and 5% CO_2_. Cleaved and full-length PARP (rabbit anti-PARP antibody from Cell Signaling and HRP-conjugated anti-rabbit antibody from Jackson ImmunoResearch) and actin (mouse anti-actin antibody from Millipore and HRP-conjugated anti-mouse antibody from Jackson ImmunoResearch), for loading control, were measured by immunoblotting.

### Determination and quantification of viable cells by flow cytometry

PC12 cells were plated overnight and incubated with three different concentrations of the various drugs for 24 hrs. To determine apoptotic and viable cells, cells were washed with PBS buffer (2 mM EDTA, 0.05% BSA in PBS) and stained with 5 µl of 7-AAD (BioLegend) for 10 min. Cells were acquired by flow cytometry (MACSQuant Analyzer, Miltenyi Biotec) and 7-AAD-negative viable cells (%) were analyzed by FlowJo software (Tree Star).

### Murine MTT cell microarray processing and analysis

Three separate MTT cell samples were prepared according to Affymetrix protocols (Affymetrix, Inc.). RNA quality and quantity was ensured using a Bioanalyzer (Agilent, Inc.) and NanoDrop (Thermo Scientific, Inc.) respectively. Per RNA labeling, 200 ng of total RNA was used in conjunction with the Affymetrix recommended protocol for GeneChip 1.0 ST chips.

The hybridization cocktail containing fragmented and labeled cDNAs was hybridized to the Affymetrix Mouse Genome ST 1.0 GeneChip. The chips were washed and stained by the Affymetrix Fluidics Station using the standard format and protocols as described by Affymetrix. The probe arrays were stained with streptavidin phycoerythrin solution (Molecular Probes, Carlsbad, CA) and enhanced using an antibody solution containing 0.5 mg/ml of biotinylated anti-streptavidin (Vector Laboratories, Burlingame, CA). An Affymetrix Gene Chip Scanner 3000 was used to scan the probe arrays. Gene expression intensities were calculated using Affymetrix AGCC software.

Partek Genomic Suite was used to RMA normalize (Robust Multichip Analysis), summarize, logtransform the data, and run ANOVA analysis and hierarchical clustering. The Series entry for the murine microarray in the NCBI Gene Expression Omnibus (GEO) database has been approaved with the following number: GSE51832 (http://www.ncbi.nlm.nih.gov/geo/query/acc.cgi?acc=GSE51832).

### Microarray data analysis

RNA extraction from MTT cells was performed as previously described [21]. RNA quality and quantity were ensured using Bioanalyzer (Agilent Technologies, Inc., Santa Clara, CA, USA) and NanoDrop (Thermo Scientific, Wilmington, DE, USA) analysis, respectively. Samples in triplicate were processed following the recommended Affymetrix protocol. Fragmented and labeled cDNA was hybridized onto Mouse GeneChip 1.0ST chip arrays (Affymetrix, Santa Clara, CA, USA). Staining of biotinylated cDNA and scanning of arrays were performed according to the manufacturer's recommendations. These data are available at the GEO database. Raw CEL-files were imported into R statistical package [22] (http://www.bioconductor.org) to perform probe set summarization, background subtraction, and quantile normalization using the RMA method. Gene-wise ***Z***-score normalization across all samples was applied to adjust for technical variation [23]. The replicate correlation was measured to be >0.98, and an average of replicates was used for further analysis.

A subset of patient data that was previously published [24] (GSE 39716) containing *SDHB* mutation samples was used for comparison with MTT cell line expression. Gene targets for the drugs tested on MTT cell lines were compiled from databases (NPC, Drug Bank and Therapeutic Target Database), and a total of 2129 genes were mapped to 22 drugs (Table S3). Datasets from mouse cell lines and SDHB samples were independently scaled to Z-scores before merging them. The merged data was subset by the common genes from drug targets, with a total of 1753 genes in common.

Genes corresponding to <2-fold change between the MTT cell line and SDHB PHEO datasets were considered for further analysis. This selection criterion resulted in a list of 1440 genes with an overall correlation of 0.86. To evaluate the similarity of drug targets between MTT vs. SDHB samples, a Pearson correlation coefficient was computed between a set of target genes from each drug with an overall average correlation of 0.8.

### Network construction, visualization and analysis

Two metabolic models corresponding to different levels of information were constructed. One is a general model that contains the 22 tested drugs and their curated target genes. The second network derives from the first one, which is based on the top 20 hub nodes. Network assembling, visualization and determination of statistical parameters were performed using Cytoscape v2.8.3 [25]. For functional and topological analysis, Reactome FI [26] and cytoHubba, plugins for Cytoscape were used. The Reactome FI plugin was designed to find network patterns related to cancer and other types of disease. This plugin accesses the Reactome FI network, a highly reliable, manually curated pathway-based protein functional interaction network covering close to 50% of human proteins, and allows construction of a FI subnetwork based on a set of genes. The cytoHubba plugin allows analysis of the topology of interaction networks and identification of essential nodes (hub nodes) that may serve as candidates of drug targets for developing novel therapies. The subnetwork of these essential nodes may help us get a more precise insight into the functions and how they collaborate together. As a measure of node importance, eccentricity scores were computed. The eccentricity is a node centrality index. The eccentricity of a node v is calculated by computing the shortest path between the node v and all other nodes in the graph, then choosing the “longest” shortest path (let (v, K) be a path, where K is the most distant node from v). Once this path with length dist(v, K) is identified, its reciprocal is calculated (1/dist(v,K)). By doing that, an eccentricity with higher value assumes a positive meaning in terms of node proximity.

**Figure 3:**
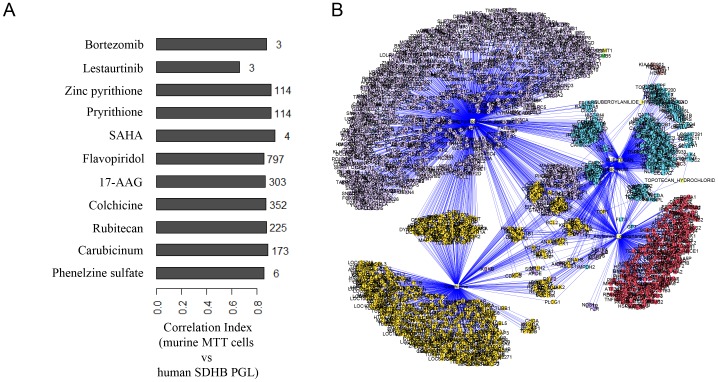
Drugs-gene targets correlation and network. A) Drug name and number of high correlated genes mapped from the human PHEO and the murine MTT microarray data set. B) Connected node network representation in which all the targets of each drug are connected to the targets of all other drugs in the network.

We have included a cytoscape network file (for figure 3B) in the Cytoscape Network S1, that can be viewed by installing the cytoscape program (http://www.cytoscape.org/download.html).

### Drug synergism analysis

For the synergism study we used the CalcuSyn Windows software for dose-effect analysis and synergism/antagonism quantification, following the manufacturer's instructions. Drug synergism was determined from median effect analysis equations developed by Chou-Talalay [27], [28]. Cell proliferation data (MTT assay) were analyzed using CalcuSyn software (Biosoft UK). Combination Index (CI) indicates *additivity* when CI = 0.8–1.2; synergism when CI<0.8; and antagonism when CI<1.2. The DRI shows the potential dose reduction of each single drug in synergistic combination at a given effect level achieved by combining these drugs.

## Results

### High-throughput screening (HTS) of the NPC Library

We screened the NIH Chemical Genomic Center Pharmaceutical Collection of clinically approved drugs, which contains 1,760 US FDA approved drugs, 785 drugs approved by other countries, 1,225 compounds in clinical trials and 56 bioactive molecules, employing a model of metastatic PHEO (by using MTT mouse PHEO cells), the most common tumor of the adrenal medulla.

The assay, in a 1536 HTS plate format, measured cell viability by determining metabolically active cells (viable cells) in culture using a luciferase, ATP-dependent readout (as described in Material and Methods), at two different time points of compound incubation (24 hrs and 48 hrs). A number of compounds showed significant cell killing, with potency below 10 µM (at 48 hrs, when the compounds showed to be more potent and efficacious). Compounds were tested at 8 doses using a quantitative HTS (qHTS) approach [20].

The qHTS identified 76 high-confidence active compounds (Table S1) with curve class (CRC) 1.1, 1.2, 2.1 and 2.2 [20], with the maximal inhibition over 60%, 3481 inactive and 269 inconclusive compounds with lower confidence CRC or showing low efficacy in the MTT cell line. Of the 76 high-confidence actives, 40 had efficacy higher than 60% and potency below 10 µM, which corresponded to a 1% hit rate from the qHTS screening. Five compounds showed significant cell killing effect with potency less than or equal to 1 µM: the protein synthesis inhibitor and antileukemic (induces apoptosis) drug homoharringtonine (IC_50_ = 0.24 µM); the tubulin inhibitors colchicine (IC_50_ = 0.47 µM), nocodazole (IC_50_ = 0.53 µM) and fenbendazole (IC_50_ = 1 µM); and the proteasome inhibitor bortezomib/velcade (IC_50_ = 0.59 µM). Table S2 shows the results of the full library of compounds, including active, inconclusive and inactive compounds. Then we performed an enrichment analysis into therapeutic categories of the compounds obtained from the primary screening. We identified that the top 5 enriched drug categories active in MTT cells were antiseptic, antimalarial, antineoplastic, estrogen and anthelmintic agents (Figure S1). However, these categories also represented the ones with fewer compounds, so, relatively, the two major categories with the most hits were represented by antineoplastic and antiseptic drugs (Figure S1). Top-ranked hits (50 compounds) based on the CRC, efficacy and potency are reported in Table 1.

**Table 1:** Top-ranked compounds (hits) with high confidence antiproliferative activity in MTT PHEO cells.

Sample Name	Curve Class	IC_50_ (µM)	Efficacy (%)
Colchicine^1^ (Colcrys)	−1.1	0.47	−85
Dipyrithione (Crimanex)	−1.1	1.50	−81
Zinc pyrithione	−1.1	2.11	−91
1-Hydroxypyridine-2-thione zinc salt	−1.1	2.11	−96
Mersalyl sodium	−1.1	2.66	−92
Auranofin (Ridaura)	−1.1	2.66	−87
Thimerosal^4^	−1.1	2.99	−87
Deslorelin acetate (Suprelorin)	−1.1	2.99	−89
Paclitaxel^1^ (Abraxane, Onxol)	−1.2	0.04	−51
5-Aza-2′-deoxycytidine, Decitabine^2^ (Dacogen)	−1.2	0.07	−52
Homoharringtonine	−1.2	0.24	−74
Trimetrexate glucuronate^5^ (Neutrexin)	−1.2	0.38	−51
Rubitecan^2^ (Orathecin)	−1.2	0.42	−55
Nocodazole^1^	−1.2	0.53	−60
Fenbendazole^1^ (Panacur)	−1.2	1.01	−61
Artemisinimum	−1.2	1.33	−60
Carmofur (Mifurol)	−1.2	1.88	−57
Suberoylanilide hydroxamic acid^2^ (SAHA) (Zolinza)	−1.2	2.37	−59
Tenovin-1	−1.2	2.66	−56
Carubicinum^2^	−1.2	2.99	−59
Captan^4^	−1.2	2.99	−66
Lissamine green B	−1.2	6.68	−54
Mycophenolic acid (CellCept, Myfortic)	−2.1	1.14	−97
Tyrothricin^4^	−2.1	1.50	−84
Mycophenolate mofetil	−2.1	1.68	−92
Brilliant Green^4^	−2.1	1.68	−108
Rotenone^5^	−2.1	2.66	−124
Lestaurtinib	−2.1	2.66	−83
Ciclopirox ethanolamine	−2.1	4.53	−85
RTA 402	−2.1	6.68	−108
Sanguinarine	−2.1	8.41	−88
Proflavine hemisulfate^2^	−2.1	8.41	−92
Parthenolide	−2.1	8.41	−104
Bortezomib^3^ (Velcade)	−2.2	0.60	−75
Albendazole^1^ (Albenza)	−2.2	0.72	−58
Sobuzoxane^2^ (Perazolin)	−2.2	0.84	−56
Azacitidine^2^ (Vidaza)	−2.2	1.33	−71
Tiquizium bromide	−2.2	1.88	−52
Flavopiridol hydrochloride hydrate	−2.2	1.88	−71
Ancitabina	−2.2	2.37	−68
Ethaverine hydrochloride	−2.2	3.60	−60
2,2′,4′-Trichloroacetophenone	−2.2	3.76	−71
Berberine chloride	−2.2	4.22	−54
17-Allylamino-geldanamycin (17-AAG)	−2.2	4.22	−81
Proguanil hydrochloride^5^	−2.2	4.53	−60
Topotecan hydrochloride^2^ (Hycamtin)	−2.2	4.73	−90
Phenelzine sulfate	−2.2	5.08	−53
Oxapium iodide	−2.2	6.68	−60
Methyl violet^4^	−2.2	7.50	−70
Malachite Green Oxalate	−2.2	8.41	−61

The table illustrates the drug name, efficacy, IC_50_ (in µM) and curve class.

1 Anti-tubulin agents;

2 Drugs targeting DNA and nucleotide analogues;

3 proteasome inhibitors;

4 antimicrobial agents;

5 antimetabolite.

### Secondary screening of selected drugs

The top 50 drugs considered active compounds based on the primary screening were grouped and mostly classified in 5 main functional categories: 1) antitubulin agents; 2) drugs targeting DNA and nucleotide analogues; 3) proteasome inhibitors; 4) antimicrobial agents; 5) antimetabolite. We found that antitubulin agents, which have a prominent class effect on protein polymerization and mitotic spindle organization, were the drug category with the most entries in the top compounds in the high-confidence list (five entries, which included colchicine, paclitaxel, and several –azoles drugs). Another class effect was represented by topoisomerase inhibitors (including carubicinum, rubitecan, sobuzoxane and topotecan), which have a prominent effect on DNA replication and telomerase maintenance.

**Figure 1:**
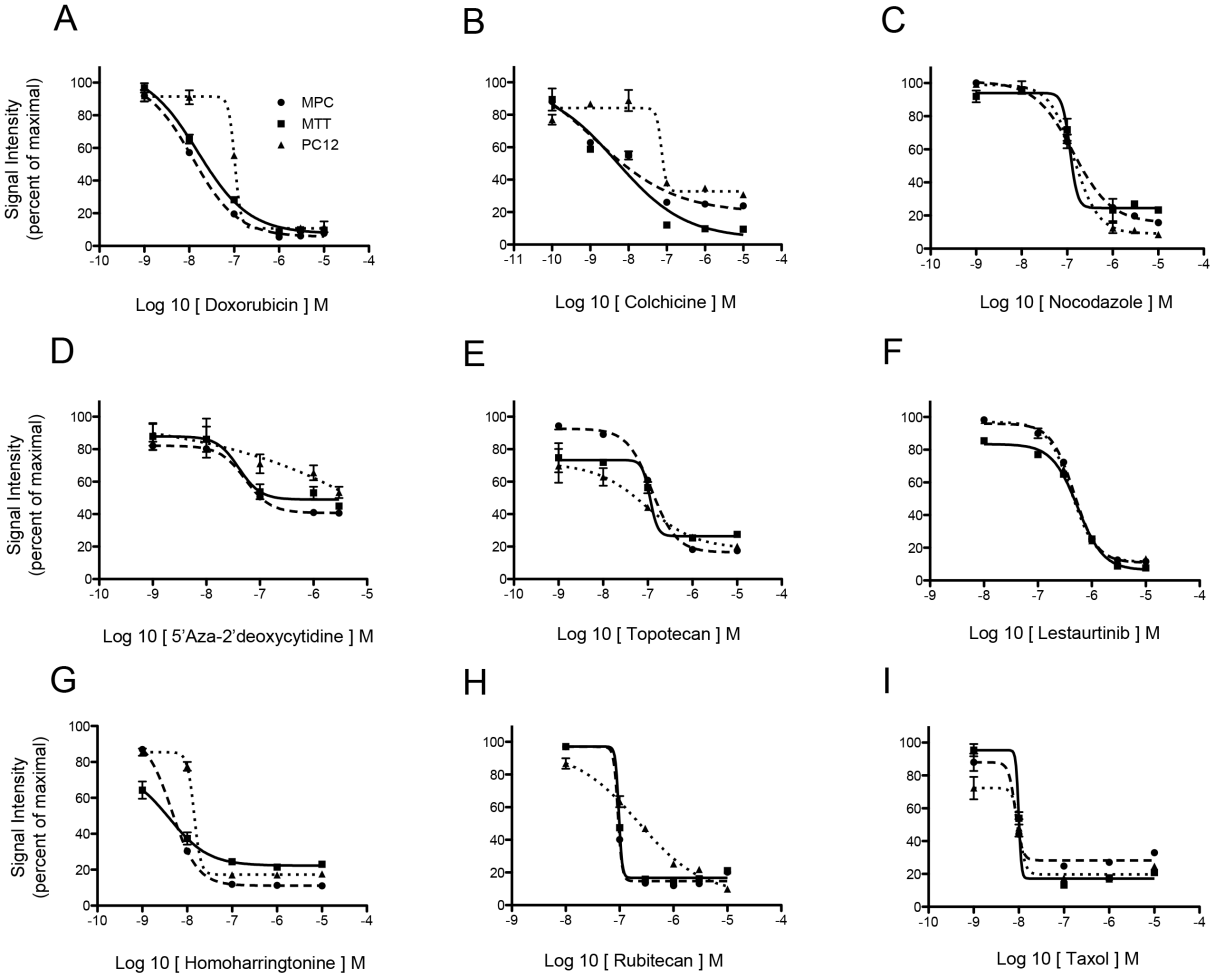
Validation of primary screening. *Panel A and B:* Dose response curves of the selected compound in MPC, MTT and PC12 PHEO cell lines. The Y-axis represents signal intensity as a percent of the maximal value. The X-axis represents the log 10 concentrations of the respective compound (inside square brackets).

A representative selection of compounds from the categories described above were selected for further investigation and validated in additional assays to confirm the activity of the hits from the HTS screen. For the secondary assays, we took advantage of a traditional proliferation assay, namely the colorimetric MTT assay, and a flow cytometric analysis for viable cells after drug treatment. We used three different PHEO cell lines, namely PC12, MPC and MTT cells (see Material and Methods for a complete description). All the compounds tested produced dose-response curves in all three cell lines, and were potent compounds having IC_50_ values in the low nanomolar range (Figure 1).

**Figure 2:**
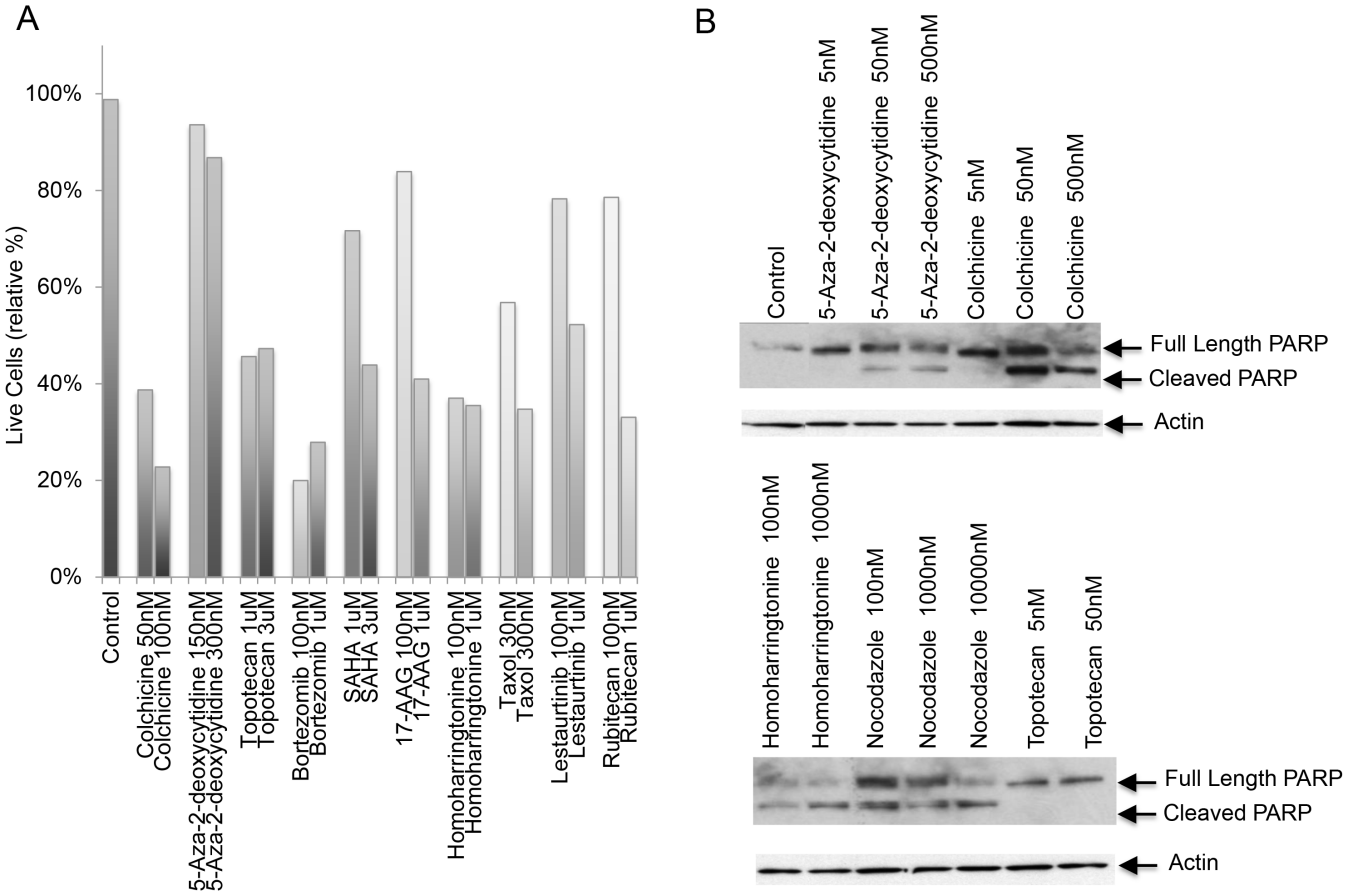
Apoptosis induced by screened drugs. A) To quantify viable cells, the membrane impermeable dye 7-amino actinomycin D (7-ADD) was added to a cell suspension of PC12 cells, after overnight treatment with the respective compound. The bar graph represents relative live cell percentage. B) MTT cells were cultured with the indicated drug and dose for 20 hr and total cell lysates were subjected to Western blot for full-length PARP, cleaved PARP and actin.

To further validate the activity of the selected compounds, we performed a flow cytometry cell viability assay using 7-AAD after overnight treatment with the selected compounds (Figure 2A). In addition, a Western blot assay was used for detection of PARP cleavage (Figure 2B) in MTT cells after 20 hr treatment. Our results from both assays from two different cell lines confirmed the activity of several antitubulin agents, including colchicine, taxol and nocodazole. Other compounds with dose-related activity included the HDAC inhibitor suberoylanilide hydroxamic acid (SAHA), the tyrosine kinase inhibitor lestaurtinib (which is an inhibitor of FLT3, JAK2, TrkA, TrkB and TrkC) and the topoisomerase inhibitor rubitecan. Furthermore, the Hsp90 inhibitor 17-AAG showed activity, confirming previously published results [29].

### Microarray data analysis and metabolic model

Because the primary and secondary screening was performed in murine cell lines (due to the unavailability of human PHEO cell lines), a strategy using microarray data from murine and human samples was used to further select a clinically relevant set of drugs from our data that could be potentially developed in human clinical trials. We first compiled a list of genes directly targeted by the top drugs discovered by our primary screening using the NPC library databank. Compounds for which information on specific targets was not available were excluded from the analysis. Based on the list of genes compiled, we selected a subset of microarray data from a new murine MTT cell microarray and a published human SDHB microarray. In particular, we focused our attention on the human microarray of patients with SDHB disease, as these patients are the ones that can benefit the most from the discovery of novel therapies. To extrapolate the murine screening data for clinical relevance, for each drug we measured the Pearson correlation coefficient between the target genes and the expression profiles from these two microarray data sets (Figure 3A). This analysis allowed us to bridge and relate the data from the murine screening with the data from an available human PHEO expression profile [24].

Two metabolic models were constructed with compounds and targets that have been compiled from our data set. The first model was constructed as a complete hypothetical network for visualization of the network assembly of 22 compounds tested on the MTT cell line (Table 2, and top compounds in Figure 3A). It contained 2153 nodes and 3091 edges (Figure 3B). All targets of a given drug were connected to all other drugs, generating a network from which maximum connected nodes can be visualized. Using eccentricity scores, a list of the top 20 targets was identified (Table S4) that were predicted to be biologically relevant. Functional enrichment analysis using Reactome Functional Interaction (FI) identified 406 gene ontology (GO) biological categories (p<0.05). Interestingly, the HDAC inhibitor SAHA (vorinostat) was one of the compounds with the highest correlation (Figure 3A) between MTT cells and human PHEO/PGL samples based on the gene profile. The second network was constructed using targets associated with GO categories (DNA replication and histone deacetylation) corresponding to two of the top hub nodes: topoisomerase and histone deacetylase, respectively. This subnetwork (Figure S2A) contained 55 nodes and 99 edges and included 5 of the 22 drug targets. Based on eccentricity analysis, carubicinum, rubitecan and colchicine (Table S5) were found to have a greater influence on the subnetwork.

**Table 2:** Combination of SAHA and epirubicin in MTT cells.

IC_50_ folds	EPI/SAHA [µM]	Fraction Affected	CI	Effect	DRI EPI/SAHA
**4×**	20/4000	0.808566317	0.719	Synergistic	8.227/1.674
**2×**	10/2000	0.740589432	0.537	Synergistic	8.593/2.378
**1×**	5/1000	0.588437471	0.568	Synergistic	5.459/2.6
**0.5**	2.5/500	0.446667935	0.553	Synergistic	4.23/3.156
**0.25**	1.25/250	0.328056979	0.518	Synergistic	3.675/4.069

CI = Combination index. DRI = Dose reduction index.

### An example of drug combination with synergism analysis

The compiled list of drugs derived from our screening represented a useful selection of compounds with the potential to work effectively in the treatment of metastatic PHEO/PGL, either as single drugs or in combination. Moreover, our combined analysis of the drug screening and the microarray data sets suggested possible drug combinations that are supported by sporadic, but useful, reports in the literature on similar drugs or drug categories in the treatment of metastatic PHEO/PGL patients.

**Figure 4:**
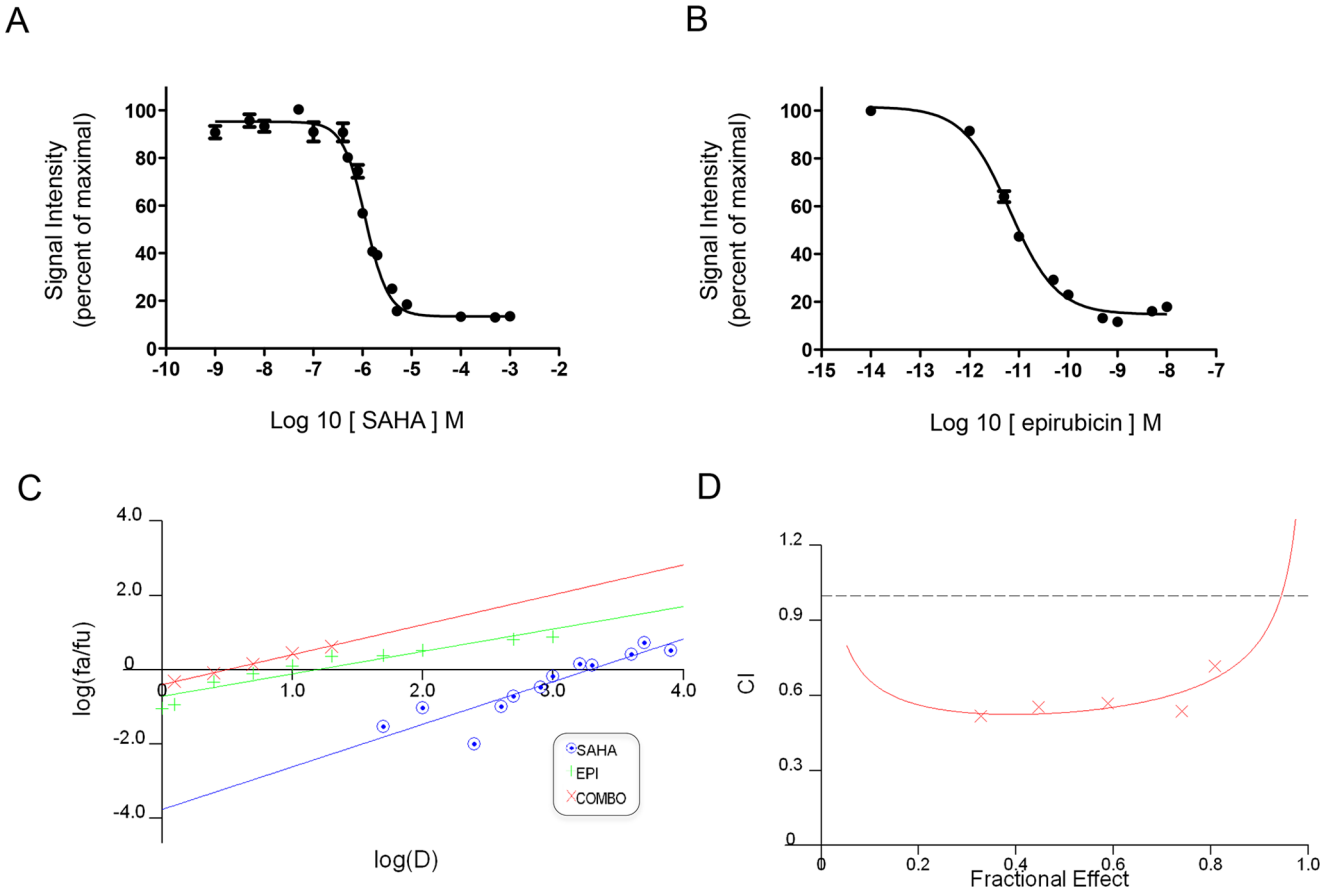
Drug combination analysis. A and B) Dose response curves for SAHA and Epirubicin; C) Median-effect plot; D) Algebraic estimate of the combinational index (CI) for the combination of epirubicin with SAHA relative to the fraction of affected cells.

The subnetwork represented in Figure S2A and B constitutes a potential source of relevant drug combinations because of the potential biological relationship between any of two nodes in the network. As an example, we selected for study the combination of the HDAC1/2 inhibitor SAHA and a structural analogue of carubicinum, namely epirubicin, which is a more clinically relevant compound based on the reported use in therapeutic combination for PHEO [30] and for other tumors [31]. We combined epirubicin with SAHA at a constant ratio (1∶200) in doses approximately equal to their IC_50_ and also in concentrations equally distributed above and below the relative IC_50_s. For this reason we first determined the relative IC_50_ for each single compound: 1 µM for SAHA and 5 nM for epirubicin (Figures 4A and B). As shown in Table 2 and Figure 4C (median-effect index) and D (algebraic estimate of the combination index), this combination produced a synergistic effect at all concentrations tested. The benefits of combining these two drugs were seen in the dose reduction index (DRI), as described in Materials and Methods. Reducing the concentration of a single drug when given in combination may lead to a reduction of the side effects of each drug. This strategy represents a promising approach to test potentially clinically useful combinations, using a combination of traditional qHTS screening testing with innovative microarray analysis.

## Discussion

In the present study, we identified drugs by a repurposing approach from the screening of an extensive library containing FDA-approved compounds that are cytotoxic for PHEO cell lines. We further validated active compounds from the screen and used data from human and murine microarrays to extrapolate potential clinically useful information to help select the hits with highest clinical potential. We used our approach to suggest an example of a synergistic drug combination using the HDAC inhibitor SAHA and the topoisomerase inhibitor epirubicin, which could represent an example of possible successful treatment for metastatic PHEO/PGL.

HTS of chemical libraries is a powerful generator of potentially clinically useful compounds to treat metastatic PHEO/PGL. This approach is novel for the field of PHEO/PGL, which is now experiencing a renaissance of interest in drug discovery, in particular in the evaluation of novel targeted therapies, especially following the discovery of several novel gene mutations (and consequently several intracellular signaling pathways with potential cellular targets) predisposing to the disease [32].

Compared with other cancer types, one of the major challenges in the development of novel therapeutics for metastatic PHEO/PGL is the absence of *bona fide* human cell lines for cell-based preliminary testing; viable and functionally active primary cells can be obtained from donated tumor tissue but unfortunately do not proliferate [33]. For this reason we used an alternative approach to extract clinically useful information by crossing and combining data from our primary qHTS drug screening with information available about the expression profiles of a murine and a human microarray data set. Of note, however, the cell lines we used in our assays represented viable models of PHEO. For example, the recent discovery of mutations in the MAX gene (which is part of the Myc-Max-Mxd1 network) [17] highlights the value of the rat PHEO cell line PC12, which lacks a functional MAX gene, as previously described [34], [35]. Also NF1 loss of function, found in the MPC cell line used in our assays, has been recently identified as a frequent occurrence in sporadic PHEO [18].

Using meta-analysis information from molecular targets of the top drugs identified by our screening with gene expression data from human and murine microarrays, we identified potential drugs to be used as single drugs or in combination.

There are multiple advantages of screening small molecule libraries of clinically approved compounds by repurposing strategies, including the possibility of introducing these drugs more rapidly into clinical trials because those drugs have already been used in humans for the treatment of other diseases, including other types of cancer. Drugs that received regulatory approval have already proven to be safe and effective for a particular disease, and we have historical information regarding the long-term side effects of the drug in a large population, which makes repurposing them for other diseases less time-consuming. Moreover this approach overcomes several of the economic and technical bottlenecks that are inherent to the drug discovery process [36]. Furthermore, the repurposing of drugs for new indications, including rare diseases such as PHEO/PGL, is gaining momentum in several areas of medicine, as the cost of developing novel chemical entities is becoming extremely expensive and time-consuming, often with a questionable outcome for the patients [11], [37].

Our primary qHTS screening has identified several compounds with potential activity on PHEO/PGL. The repertoire of compounds identified is a potential source available to the PHEO/PGL community for further testing, either as single drugs or in combination, either with other drugs in our list or with other drugs that have shown activity on PHEO. Of particular interest, the antitubulin drugs seem to emerge from our screening as a group of drug with a prominent “class effect” on PHEO, with potent activity in the low nanomolar range. This suggests that these compounds may have activity as single agents in the treatment of metastatic PHEO and possibly PGL.

The secondary screening confirmed activity of several compounds, including the antitubulin agents, the HDAC inhibitor SAHA and the topoisomerase inhibitor rubitecan. Subsequent analysis combining the results from the screening with data from expression microarray assays resulted in the selection of two drugs for combination testing.

The drug combination we chose as an example of using data from the human microarray crossed with the cellular network of gene targets of the drug identified by the screening find validation also in the literature. Interestingly, SAHA has been described as a drug that induces SDHB protein stabilization and the entry of this protein (although mutated but still functional) into the mitochondria [38]. HDAC inhibitors were also found to be excellent drugs to increase the uptake of MIBG into PHEO cells [39]. Moreover, the anthracycline drug epirubicin has been already used with success in a drug combination study for the treatment of malignant PHEO [30]. The combination of topoisomerase and HDAC inhibitors has been explored also in other cancer types. Recently, Gray et al. [31] described this combination in small cell lung cancer, and other reports validate this therapeutic approach. The optimal dosing and timing of administration is still an open debate in the literature, and future experiments will need to address this problem in the setting of PHEO. Interestingly, another topoisomerase II inhibitor, doxorubicin, that was used as a control in the qHTS screening and in the MTT assays, showed good activity in inhibiting the proliferation of PHEO cell lines. In addition, HDAC inhibitors may influence the level of acetylation of other non-histone effector molecules, including Hsp90 and NF-kB, two other important molecular targets in PHEO.

Of note, our screening was able to identify compounds that we have already explored for the potential treatment of PHEO, and so they acted as internal controls of the validity of our approach. For example, one of the drugs in the top 50 active compound list was the Hsp90 inhibitor 17-AAG, which we investigated in further detail in another study [29]. Two other compounds, RTA 402 and parthenolide, have NF-kB as a molecular target, in agreement with our recent study on the role of its inhibition as a potential therapeutic approach [40]. Interestingly, we also found that several compounds from our screening are in the same class/category of compounds in the mainstay chemotherapy treatment (CVD combination), such as microtubule inhibitors, which represent a prominent category in our results.

Another prominent category from our screening was represented by drugs targeting DNA. Recent evidence points to a role of DNA methylation in the pathogenesis of SDH mutant PHEOs [41], opening the opportunity to use drugs such as the DNA methyltransferase inhibitor decitabine (5-aza-2′deoxycytabine), which was one of the hits in our screening.

In conclusion, we have presented here the adoption of an integrated approach to discover potentially clinically useful compounds for the treatment of metastatic PHEO/PGL, and we hope that this strategy will help to move forward the field of drug development for other orphan diseases.

## Supporting Information

Cytoscape Network S1 Supplemental data.
(CYS)

Figure S1 Enrichment analysis. Enrichment analysis (by therapeutic category) of active compounds from the primary screening of the NPC drug library. Gray bars represent the total number of drugs in the specific therapeutic category, white bars represents the number of active compounds and black bars represents the results of the enrichment analysis as described in the Material and Methods section. The green dash line marks an enrichment ratio >20% in the active drugs.
(TIF)

Figure S2 Subnetwork and hubnodes. A) Subnetwork with all interactions associated with drugs that include topoisomerase (DNA replication) and histone deacetylase (telomerase maintenance). B) Top 20 hubnodes network derived from the global network, colored by up- and down-regulated genes common to human PHEO SDHB and murine MTT cells.
(TIF)

Table S1 NPC library screening, top compounds. Seventy-six high-confidence active compounds identified in the quantitative high-throughput screening with curve class 1.1, 1.2, 2.1 and 2.2, and maximal inhibition over 60%.
(XLSX)

Table S2 NPC library screening, complete list.Complete list of compounds from the NPC library including active, inconclusive and inactive compounds. The table includes the NCG compound identification number (Sample ID), chemical structure composition, type of curve class based on the screening analysis, IC_50_ (in µM), efficacy and the identification of the compound as active, inconclusive or inactive based on the curve classification illustrated in Figure S1.
(XLS)

Table S3 Gene targets-drugs correlation. List of drugs and their targets compiled from databases as described in Materials and Methods (first column) or mapped to the microarray (second column).
(DOCX)

Table S4 Centrality scores. Top 20 genes based on the centrality score from the network common to SDHB (human) and MTT cells (murine), to look at the similarity of the dataset (MTT with SDHB) based on the microarray data.
(DOCX)

Table S5 Hubnodes scores. Top 20 hubnodes (nodes with the most association in the network) showing the node importance score (derived from the eccentricity method). The hubnode can be either a gene or a drug in the interrelating network. The highest is the number of associations of a hubnode, the highest is the score.
(DOCX)
