# Supplementary figures and images for: High-Throughput Screening for the Identification of New Therapeutic Options for Metastatic Pheochromocytoma and Paraganglioma
Source: PLoS One. 2014 Apr 3;9(4):e90458. doi: 10.1371/journal.pone.0090458 (PMC3974653; doi:10.1371/journal.pone.0090458)

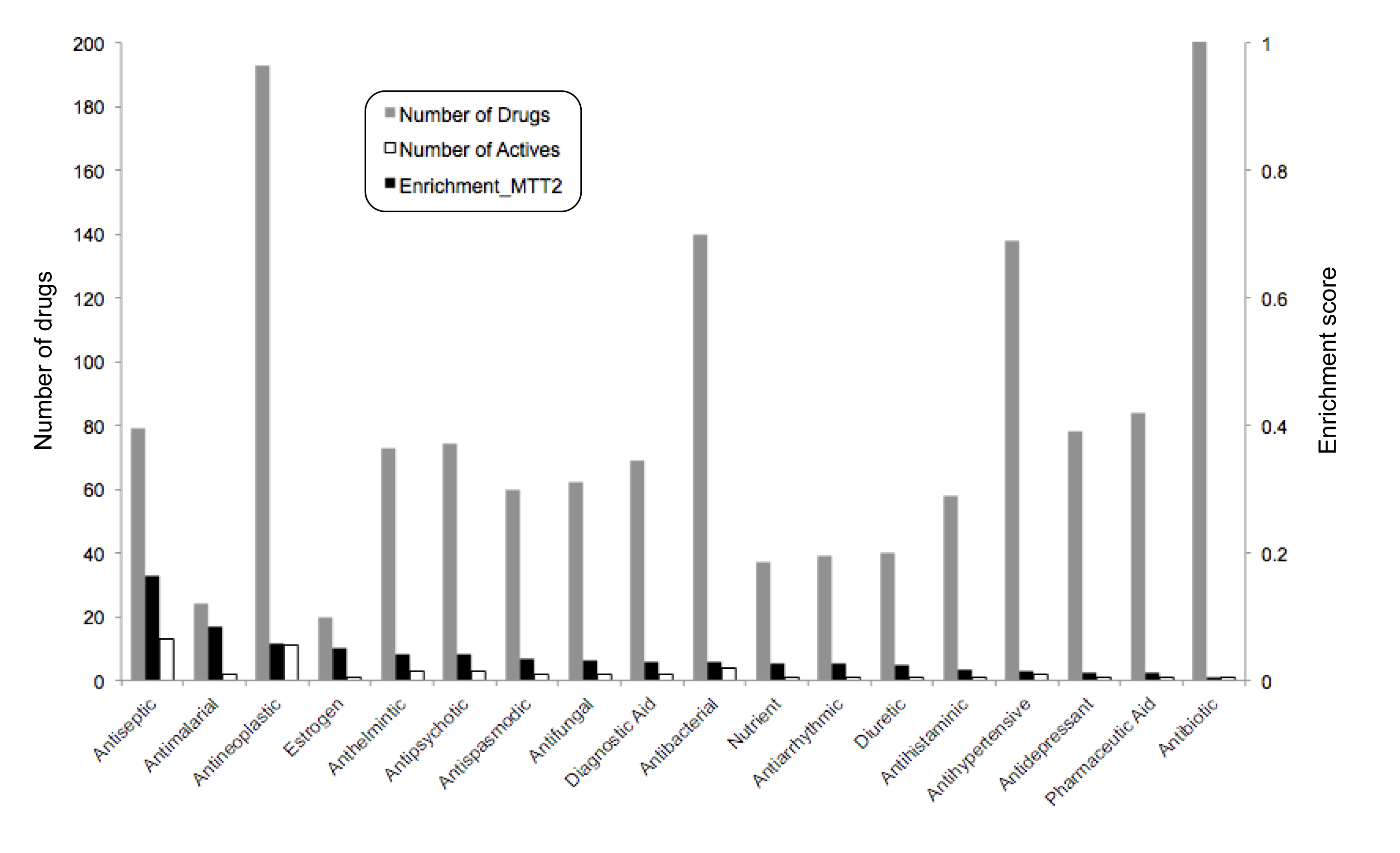

Supplement: Figure S1 — Enrichment analysis. Enrichment analysis (by therapeutic category) of active compounds from the primary screening of the NPC drug library. Gray bars represent the total number of drugs in the specific therapeutic category, white bars represents the number of active compounds and black bars represents the results of the enrichment analysis as described in the Material and Methods section. The green dash line marks an enrichment ratio >20% in the active drugs. (TIF) [file pone.0090458.s002.tif]

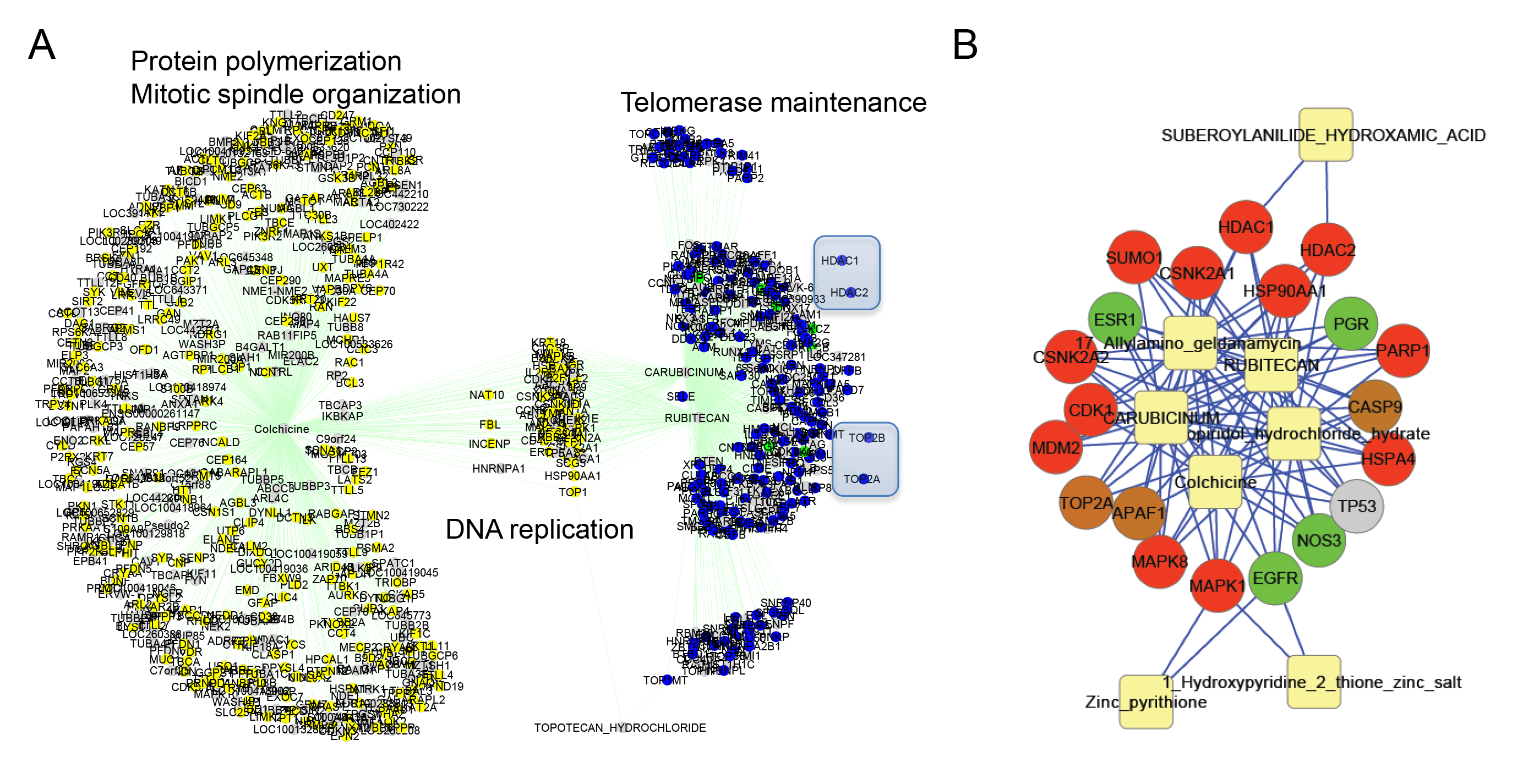

Supplement: Figure S2 — Subnetwork and hubnodes. A) Subnetwork with all interactions associated with drugs that include topoisomerase (DNA replication) and histone deacetylase (telomerase maintenance). B) Top 20 hubnodes network derived from the global network, colored by up- and down-regulated genes common to human PHEO SDHB and murine MTT cells. (TIF) [file pone.0090458.s003.tif]
