# Supplementary material for: High-Throughput Screening for the Identification of New Therapeutic Options for Metastatic Pheochromocytoma and Paraganglioma
Source: PLoS One. 2014 Apr 3;9(4):e90458. doi: 10.1371/journal.pone.0090458 (PMC3974653; doi:10.1371/journal.pone.0090458)
Supplement: Table S3 — Gene targets-drugs correlation. List of drugs and their targets compiled from databases as described in Materials and Methods (first column) or mapped to the microarray (second column). (DOCX) [file pone.0090458.s006.docx]

**Table S3.**

| **Name** | **Compiled from database** | **Mapped to array** |
| --- | --- | --- |
| Flavopiridol-hydrochloride-hydrate | 1146 | 983 |
| Colchicine | 550 | 425 |
| 17-Allylamino-geldanamycin | 406 | 361 |
| Rubitecan | 335 | 301 |
| Carubicinum | 266 | 237 |
| 1-Hydroxypyridine-2-thione-zinc-salt | 159 | 136 |
| Zinc pyrithione | 159 | 136 |
| Phenelzine-sulfate | 14 | 7 |
| SAHA | 10 | 4 |
| Bortezomib | 9 | 4 |
| Lestaurtinib | 5 | 5 |
| Mycophenolate mofetil | 5 | 1 |
| Mycophenolic acid | 5 | 1 |
| Auranofin | 5 | 2 |
| Azacitidine | 4 | 1 |
| 5-Aza-2-deoxycytidine | 4 | 1 |
| Topotecan hydrochloride | 2 | 2 |
| Proflavine hemisulfate | 2 | 1 |
| Paclitaxel | 2 | 2 |
| Proguanil hydrochloride | 1 | NA |
| Parthenolide | 1 | NA |
| Trimetrexate glucuronate | 1 | NA |
