# Supplementary material for: High-Throughput Screening for the Identification of New Therapeutic Options for Metastatic Pheochromocytoma and Paraganglioma
Source: PLoS One. 2014 Apr 3;9(4):e90458. doi: 10.1371/journal.pone.0090458 (PMC3974653; doi:10.1371/journal.pone.0090458)
Supplement: Table S4 — Centrality scores. Top 20 genes based on the centrality score from the network common to SDHB (human) and MTT cells (murine), to look at the similarity of the dataset (MTT with SDHB) based on the microarray data. (DOCX) [file pone.0090458.s007.docx]

**Table S4.**

| Rank | Name | Score |
| --- | --- | --- |
| 1 | TP53 | 0.24919 |
| 1 | PARP1 | 0.24919 |
| 1 | HDAC2 | 0.24919 |
| 1 | SUMO1 | 0.24919 |
| 1 | APAF1 | 0.24919 |
| 1 | TOP2A | 0.24919 |
| 1 | CDKN1A | 0.24919 |
| 1 | CASP9 | 0.24919 |
| 1 | CDK1 | 0.24919 |
| 1 | MAPK8 | 0.24919 |
| 1 | HDAC1 | 0.24919 |
| 1 | MAPT | 0.24919 |
| 1 | POLR2A | 0.24919 |
| 1 | FGF1 | 0.24919 |
| 1 | CASP3 | 0.24919 |
| 1 | RAD51 | 0.24919 |
| 1 | EGFR | 0.24919 |
| 1 | BIRC5 | 0.24919 |
| 1 | MDM2 | 0.24919 |
| 1 | MAPK1 | 0.24919 |
