# Supplementary material for: High-Throughput Screening for the Identification of New Therapeutic Options for Metastatic Pheochromocytoma and Paraganglioma
Source: PLoS One. 2014 Apr 3;9(4):e90458. doi: 10.1371/journal.pone.0090458 (PMC3974653; doi:10.1371/journal.pone.0090458)
Supplement: Table S5 — Hubnodes scores. Top 20 hubnodes (nodes with the most association in the network) showing the node importance score (derived from the eccentricity method). The hubnode can be either a gene or a drug in the interrelating network. The highest is the number of associations of a hubnode, the highest is the score. (DOCX) [file pone.0090458.s008.docx]

**Table S5.**

| Rank | Name | Score |
| --- | --- | --- |
| 1 | Carubicinum | 0.33333 |
| 1 | Rubitecan | 0.33333 |
| 1 | Colchicine | 0.33333 |
| 4 | LIG4 | 0.25 |
| 4 | 17-Allylamino-geldanamycin | 0.25 |
| 4 | KRT7 | 0.25 |
| 4 | TOP1 | 0.25 |
| 4 | HDAC9 | 0.25 |
| 4 | SSRP1 | 0.25 |
| 4 | CHEK1 | 0.25 |
| 4 | RAD9A | 0.25 |
| 4 | CDC25C | 0.25 |
| 4 | HMGB2 | 0.25 |
| 4 | HDAC2 | 0.25 |
| 4 | HDAC6 | 0.25 |
| 4 | RMI1 | 0.25 |
| 4 | Flavopiridol hydrochloride hydrate | 0.25 |
| 4 | EGF | 0.25 |
| 4 | TYMS | 0.25 |
| 4 | RFC4 | 0.25 |
